# Monomeric bile acids modulate the ATPase activity of detergent-solubilized ABCB4/MDR3

**DOI:** 10.1016/j.jlr.2021.100087

**Published:** 2021-05-20

**Authors:** Tim Kroll, Sander H.J. Smits, Lutz Schmitt

**Affiliations:** Institute of Biochemistry, Heinrich Heine University Düsseldorf, Düsseldorf, Germany

**Keywords:** ABC transporter, ABCB4/MDR3, bile acids, cholesterol, ATPase activity, TLCA, Nor-UDCA, critical micelle concentration, ABC, ATP binding cassette, BAM, bile acid mixture, BSEP, bile salt export pump, CA, cholic acid, CDCA, chenodeoxycholic acid, CHAPS, 3-[(3-cholamidopropyl)dimethylammonio]-1-propanesulfonate, CHAPSO, 3-[(3-cholamidopropyl)dimethylammonio]-2-hydroxy-1-propanesulfonate, DCA, deoxycholic acid, DOPC, 1,2-dioleoyl-sn-glycero-3- phosphocholine, G/T/CA, glyco-/tauro-/cholic acid, G/T/CDCA, glyco-/tauro-/chenodeoxycholic acid, G/T/DCA, glyco-/tauro-/deoxycholic acid, G/T/UDCA, glyco/-tauro-/ursodeoxycholic acid, LCA, lithocholic acid, MDR3, multidrug-resistant protein 3, NBD, nucleotide binding domain, NTCP, sodium taurocholate transporting peptide, PC, phosphatidylcholine, TCA, taurocholic acid, TCDCA, taurochenodeoxycholic acid, TLCA, taurolithocholic acid, TMD, transmembrane domain, UDCA, ursodeoxycholic acid

## Abstract

ABCB4, also called multidrug-resistant protein 3 (MDR3), is an ATP binding cassette transporter located in the canalicular membrane of hepatocytes that specifically translocates phosphatidylcholine (PC) lipids from the cytoplasmic to the extracellular leaflet. Due to the harsh detergent effect of bile acids, PC lipids provided by ABCB4 are extracted into the bile. While it is well known that bile acids are the major extractor of PC lipids from the membrane into bile, it is unknown whether only PC lipid extraction is improved or whether bile acids also have a direct effect on ABCB4. Using in vitro experiments, we investigated the modulation of ATP hydrolysis of ABC by different bile acids commonly present in humans. We demonstrated that all tested bile acids stimulated ATPase activity except for taurolithocholic acid, which inhibited ATPase activity due to its hydrophobic nature. Additionally, we observed a nearly linear correlation between the critical micelle concentration and maximal stimulation by each bile acid, and that this modulation was maintained in the presence of PC lipids. This study revealed a large effect of 24-nor-ursodeoxycholic acid, suggesting a distinct mode of regulation of ATPase activity compared with other bile acids. In addition, it sheds light on the molecular cross talk of canalicular ABC transporters of the human liver.

Human bile, which is formed at the canalicular membrane of hepatocytes, consists mainly of mixed micelles formed by predominately phosphatidylcholine (PC) lipids, bile acids, and cholesterol as well as to a smaller extent bilirubin, glucuronides, and organic anions. All these molecules are transported into the bile canaliculi by different ABC transporters. Bile salts are secreted by ABCB11 (bile salt export pump [BSEP]) (1), PC lipids are translocated by ABCB4 (2) and cholesterol is the substrate of the heterodimeric ABC transporter ABCG5/G8 (3). Furthermore, ABCC2 (MRP2) excretes bilirubin (4) and glucuronidated metabolites (5, 6). Further information concerning human hepatobiliary ABC transporter is summarized in (7). In general, the major component of bile is bile acids, which make up approximately 70% of human gallbladder bile (8). In humans, four different bile acids are present (Fig. 1A–D), which can be divided into primary and secondary bile acids. Primary bile acids are derived from cholesterol and are synthesized in hepatocytes by a two-step pathway resulting in either the trihydroxy cholic acid (CA) (Fig. 1A) or the two hydroxy chenodeoxycholic acid (CDCA) (Fig. 1B) (9, 10, 11, 12, 13). For higher solubility both are conjugated with either glycine or taurine. In humans, the major conjugation is glycine (8, 11, 12). In rodents (e.g., mouse or rats), however, taurine is the main conjugate. These conjugated primary bile acids are then secreted via ABCB11 into bile, stored in mixed micelles, until bile is secreted into the intestine. There, bile acids are required to solubilize hydrophobic compounds, e.g., vitamins or fatty acids. However, the bile acids themselves remain not unmodified in the intestine. Intestinal bacteria modify the conjugated primary bile acids by deconjugation and dehydroxylation specifically at position 7 (14). This results in so-called secondary bile aids. The two hydroxy deoxycholic acid (DCA) (Fig. 1C) is derived from CA, while the monohydroxy lithocholic acid (LCA) (Fig. 1D) is derived from CDCA. Through these modifications, the bile pool becomes more hydrophobic. Next reabsorption (active or passive) results in uptake of nearly 95% of the bile acids from the ileal segment into the blood (15), where it is transported back in to the liver. Here, the sodium taurocholate transporting peptide (NTCP) takes up primary and secondary bile acids (16). This circulation of bile acids is called “enterohepatic circulation.” For detailed information, we recommend the review of Martinez-Augustin (17). Furthermore, next to the four bile acids described above, two nonhuman bile acids are relevant in medical treatments. Ursodeoxycholic acid (UDCA) (Fig. 1E) is part of the Chinese black bear bile pool and possesses the highest similarity to CDCA. The only but important difference is the stereochemistry of the hydroxy group at position 7. While the hydroxy group at position 7 (if present) in all human bile acids is in the alpha position, the hydroxy group of UDCA is in the beta position (18). UDCA is a common drug in the treatment of cholestatic liver diseases (19), such as primary biliary cirrhosis (20, 21, 22), intrahepatic cholestasis of pregnancy (23), or progressive familial intrahepatic cholestasis (24, 25, 26). Since UDCA is such a powerful and widely distributed drug, it was also the target of synthetic modifications. These investigations resulted in a side-chain shortened derivate, 24-*nor*-ursodeoxycholic acid (Nor-UDCA). In comparison to UDCA, it lacks a methylene unit of its side chain (Fig. 1F). This minimal chemical modification results in resistance to amidation with taurine or glycine compared with UDCA (27). Additionally, Nor-UDCA does not undergo the entire enterohepatic circulation, instead it undergoes cholehepatic shunting, which represents the reabsorption by cholangiocytes (28). Nor-UDCA is seen as a novel approach in cholestatic and metabolic liver diseases (29, 30). In the case of ABCB4 knockout mice, Nor-UDCA was superior to UDCA in the treatment of sclerosing cholangitis (31, 32).

**Fig. 1:**
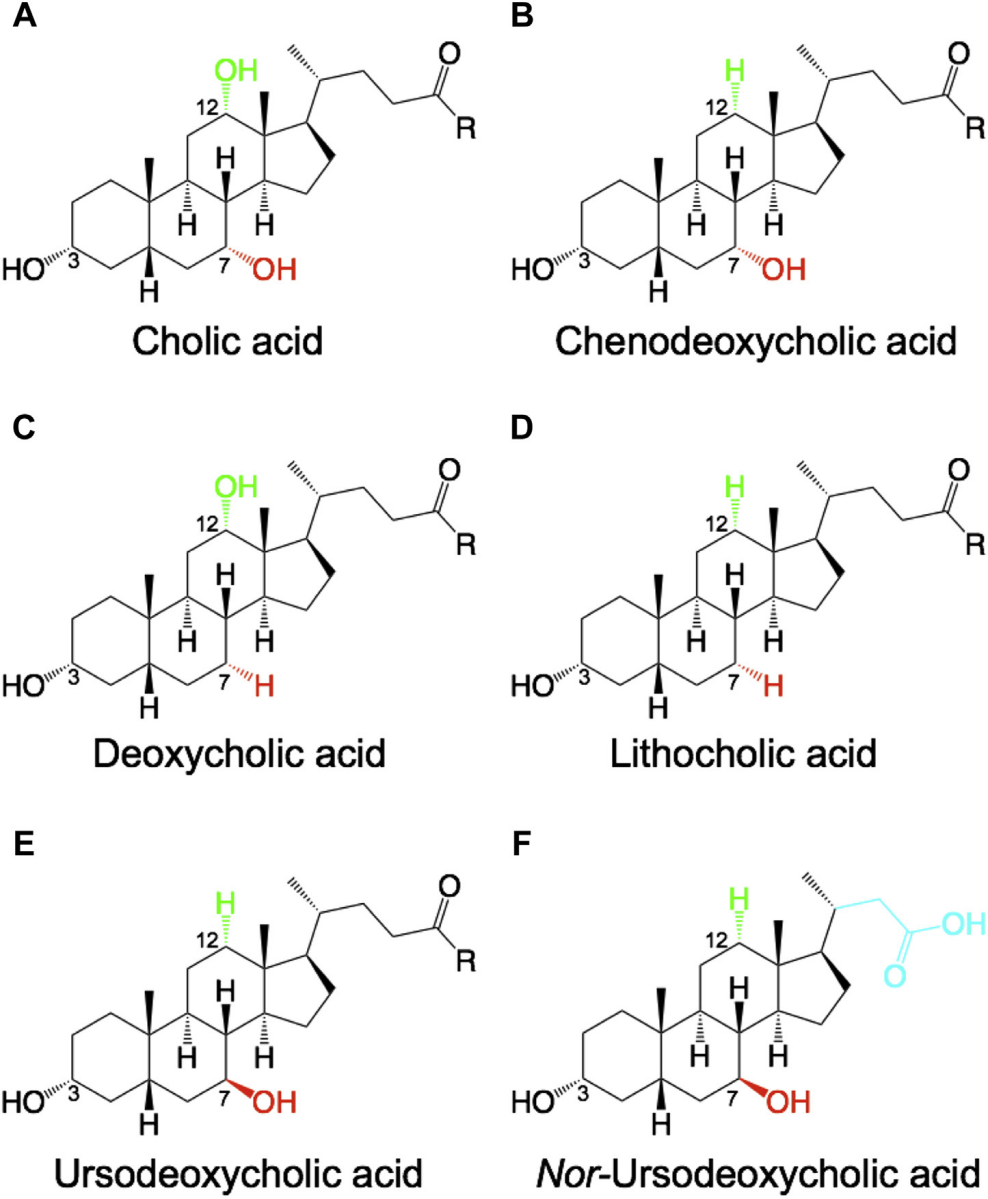
Chemical structure of relevant bile acids. Bile acids are unconjugated (R equals a hydroxyl group) after synthesis, but get conjugated with either glycine or taurine (R equals glycine or taurine) prior to transport. The primary bile acids cholic (A) and chenodeoxycholic (B) acid are synthesized by two different pathways resulting in either a hydroxylation at position 12 for cholic acid or no hydroxylation at position 12 for chenodeoxycholic acid. Secondary bile acids are derived from the primary ones by dehydroxylation at position 7 by bacteria in the ilium. While deoxycholic acid (C) derives from cholic acid (A) lithocholic acid (D) is the result of dehydroxylation of chenodexycholic acid (B). Ursodeoxycholic acid (E) can be found in small amounts in human, but is one of the major bile acids in Chinese black bear (*Ursus thibetanus*). *Nor-*Ursodeoxycholic acid (F) in comparison to UDCA lacks a methylene group in its side chain (highlighted in violet). Therefore, it is not conjugated like the other bile acids.

ABCB4 belongs to the superfamily of ATP binding cassette (ABC) transporters. The membrane proteins within this superfamily are present in all three kingdoms of life and share a common blueprint (33, 34). In the human genome, 48 genes coding for ABC transporter have been identified, which are divided into seven subfamilies (35). ABCB4 is part of the subfamily B and consists of two transmembrane domains (TMDs) and two nucleotide binding domains (NBDs) encoded on one single gene. Thus, ABCB4 represents a so-called full-size transporter (36), the structure of which was determined in 2019 (37). Due to the high identity (76%) and similarity (86%) of ABCB4 to the P-glycoprotein (P-gp, ABCB1), also termed multidrug resistance protein 1 (MDR1), ABCB4 was termed MDR3. In contrast to the ubiquitous expressed P-gp, ABCB4 is only expressed in hepatocytes (38, 39) and specifically targeted to the apical (canalicular) membrane. Additionally, ABCB4 possesses a different function as P-gp. First indications were derived from the murine homologue of ABCB4, Mdr2. Homozygous *mdr2*^*−/−*^ knockout mice lacked cholesterol and PC lipids in their bile (2). Complementation of mice *mdr2*^*−/−*^ by human ABCB4 demonstrated that human ABCB4 carried out the same function as Mdr2 (40). Finally, it was shown that ABCB4 specifically recognizes PC lipids (41, 42, 43). Nevertheless, data demonstrated that ABCB4 recognized certain P-gp substrates and inhibitors (44, 45). Based on these findings, it is now generally accepted that in vivo ABCB4 specifically translocates lipids of the PC family from the inner to the outer leaflet of the canalicular membrane of hepatocytes and therefore is part of the bile triumvirate. This bile triumvirate is composed of ABCB4, the bile acid export pump ABCB11 (or BSEP), and the cholesterol heterodimeric transporter ABCG5/G8. Since the substrates of this bile triumvirate are the major compounds of primary bile and form mixed micelles, one might speculate that these three ABC exporters act in concert to ensure proper formation of bile and balance of their compounds (46). For ABCB11, it was demonstrated that the amount of membrane cholesterol has an effect on its activity (47). Furthermore, it was shown for detergent-purified ABCG5/G8 that bile acids stimulate ATPase activity in a concentration-dependent manner (48). For ABCB4, it is known that the presence of a bile acid such as taurocholic acid (TCA) can increase the PC lipid and cholesterol content in the extracellular medium (49). For example, studies with murine ABCB4 demonstrated an explicit higher PC lipid content in mouse bile in the presence of TCA compared with the situation in the absence of TCA (50). In line with these findings is a preferential release of PC lipids from rat liver canalicular vesicles in the presence of TCA (51). For human ABCB4 expressed in HEK cells, it was demonstrated that adding TCA to the extracellular medium results in an increased amount of PC lipid and cholesterol in the extracellular medium after 24 h (49). Furthermore, in a model cell line (LLC-PK_1_) expressing all three ABC transporters involved in bile formation, higher NBD-labeled PC lipid concentrations in the medium were observed, if cells were treated with albumin or TCA (52). These assays however cannot distinguish between a direct effect of the bile acid on ABCB4 or whether only PC lipid extraction is improved (37).

In this study, we aimed to address this question by measuring the ATPase activity of ABCB4 under defined conditions. The rationale behind this is the coupling between ATP hydrolysis and PC translocation, i.e., higher ATPase activity is the prerequisite for more efficient PC transport. Therefore, we used the previously established heterologous expression system in *Picheria pastoris*, which allows the purification of human ABCB4 (53), which enabled the determination of the kinetic parameters of the basal ATPase activity of ABCB4 wild type (54). In the same study, we were able to demonstrate that lipids of the PC family such as 1,2-dioleoyl-sn-glycero-3-phosphocholine (DOPC) stimulated ATPase activity of detergent-purified ABCB4, while such a stimulation did not occur for non-PC lipids (54). This clearly reflects the in vivo situation (40, 41, 42, 55, 56). In this study, we now used this established system to investigate the effect of conjugated or unconjugated human bile acids on the ATPase activity of human ABCB4 in vitro. Additionally, we analyzed conjugated and unconjugated UDCA and Nor-UDCA due to their clinical relevance as well as cholesterol as natural part of the canalicular membrane and substrate of ABCG5/G8. To evaluate our results quantitatively, we also determined the critical micelle concentration (cmc) of the bile acids used in our study under the conditions of our assay. Additionally, the observed modulation of ATPase activity of ABCB4 by bile acids and cholesterol was put in the context of a possible competition between bile acids and PC lipids. Here, we demonstrate that bile acids below their cmc actually modulate the ATPase activity of ABCB4 in a concentration-dependent manner. This effect occurred also in the presence of a natural substrate, DOPC, or cholesterol. This clearly demonstrates that all three substrates of all three bile triumvirate ABC transporters effect the function of ABCB4.

## Materials and methods

### Chemicals

Fos-choline 16 (FC-16) was obtained from Anatrace and DOPC from Avanti Polar Lipids. All bile acids were purchased from Merck except the conjugated versions of UDCA, Nor-UDCA, which was provided by Prof. Dr Dieter Häussinger, University Hospital Düsseldorf.

### Routine procedures

Protein concentration was detected by the Bradford Coomassie Plus Assay (Pierce) or by measuring the absorbance at 280 nm using a NanoDrop™ 1000 Spectrometer (Thermo Fisher Scientific). The monoclonal anti-P-gp C219 antibody (Merck) combined with an anti-mouse IgG-HRP conjugate (Dianova) was employed for immune detection of ABCB4.

### Expression and purification of human wild-type ABCB4

Expression, solubilization, and purification of human ABCB4 were performed as described in Kluth *et al.* (54) with the modifications outlined below. Detailed information about cloning and transformation was described in Stindt *et al.* (57) and Ellinger *et al.* (53). For higher cell density during fermentation, the glycerol-fed batch phase was increased to 5–6 h, therefore the expression (methanol-fed batch phase) could be reduced to 24 h as stated in (43). Solubilization was performed at 18°C (43) instead of 4°C (54) to obtain higher amounts of solubilized protein.

### BODIPY® FL maleimide labeling of purified ABCB4

ABCB4 was specifically inhibited by labeling the unique cystine of the Walker A motif as described for ABCB1 (58) and ABCB4 (54). A sample of purified ABCB4 was incubated with 10-fold molar excess of BODIPY® FL maleimide (BODIPY® FL N-(2- aminoethyl)maleimide, Molecular Probes) at 22°C for 20 min. The reaction was quenched after 20 min by addition of a 20-fold molar excess of dithiothreitol (DTT) at 22°C for 10 min. Qualitative labeling efficiency was analyzed by visualizing a sample after SDS-PAGE by UV excitation.

### Lipid, cholesterol, and bile acid preparation

DOPC was dissolved in chloroform, the solvent was evaporated, and the dried lipid sample was redissolved in ATP hydrolysis reaction buffer (50 mM Tris HCl, pH 7.4 at 37°C, 100 mM NaCl) at a concentration of 25.4 mM following the protocol of Geertsma *et al.* (59). Prior to the addition of the DOPC sample to the ATP hydrolysis assay, it was diluted to 5 mM, sonicated until a clear solution was obtained, and subsequently used at a final concentration of 300 μM. Since chloroform is not suitable for the later ATPase assay and cholesterol at higher concentration is insoluble in water and nearly all solvents, a molar mixture of cholesterol and DOPC was used. This mixture was obtained by adding the required molar ratio of cholesterol to always the same amount of dried DOPC. Afterward, the mixture was treated identical to DOPC only as described above resulting in a final concentration (f.c.) of 300 μM DOPC with 0–1,000 μM cholesterol in the ATPase assay.

The sodium salts of conjugated and unconjugated bile acids were dissolved in double-distilled water (ddH_2_O) or 100% DMSO at a concentration of 100 mM. While glyco-/tauro-/cholic acid (G/T/CA), glyco-/tauro-/chenodeoxycholic acid (G/T/CDCA) were dissolved in ddH_2_O water, glyco-/tauro-/deoxycholic acid (G/T/DCA), glyco/-tauro-/ursodeoxycholic acid (G/T/UDCA) as wells as Nor-UDCA and taurolithocholic acid (TLCA) were dissolved in DMSO. Glyco- and unconjugated lithocholic acid could not be dissolved in DMSO in the appropriate concentrations and therefore excluded. DMSO concentrations, if necessary, were set equal within one setup and did not exceed 1% for G/T/Nor-/UDCA or 5% for TLCA. Modulation of the ATP hydrolysis by bile acid was determined in a concentration-dependent manner from 0 to 1,000 μM bile acids.

### ATP hydrolysis activity measurement of purified ABCB4

Analysis of the ATP activity of ABCB4 was performed by determining the amount of released, free inorganic orthophosphate by the malachite green assay (53, 54, 60) with minor modifications. The assay was performed in a total volume of 100 μl consisting of reaction buffer (50 mM Tris HCl, pH 7.4 at 37°C, 100 mM NaCl) supplemented with the 2.5 × cmc of FC-16 and 10 mM MgCl_2_. For determining the effect of DOPC, different bile acids or combinations thereof, 1–5 μL of the corresponding stock solutions (see lipid and bile acid preparation) were added. Five micrograms of ABCB4 or the BODIPY®-labeled sample was used in every reaction sample. Reaction was started by addition of ATP (5 mM f.c.) and performed for 40 min at 37°C. After 0 and 40 min, the reaction was stopped by transferring 25 μl of the reaction mixture into a 96-well plate containing 175 μl of 20 mM ice-cold sulfuric acid. Additionally, a phosphate standard with concentrations raging from 0 to 500 μM was used. Subsequently, inorganic phosphate was stained by adding 50 μl of dye (0.096% (w/v) malachite green, 1.48% (w/v) ammonium molybdate, 0.173% (w/v) Tween-20, and 2.36 mM sulfuric acid). After incubation for 15 min, the absorption at 595 nm was measured (iMark™ Microplate Reader, BioRad), and the concentrations of phosphate release were calculated based on the slope of the line calculated based on the phosphate standards.

Basal ATPase activity of ABCB4 was set to 0% and the effect of supplements was calculated as % stimulation or % inhibition of the basal activity, respectively. For determination of kinetic parameters, bile acid concentration was varied and analyzed according to an allosteric sigmoidal fit (Equation 1):

(1) $$v=\;\frac{v_{max}*{\left[S\right]}^{h}}{EC_{50}^{h}+\;{\left[S\right]}^{h}}$$

Here, v describes the stimulation (%), v_max_ is the maximal stimulation (%), EC_50_ represents the half maximal effective concentration, S is the substrate concentration, and h is the Hill coefficient

In case of inhibition, the reduction (%) was plotted against the bile acid concentration and data were analyzed using Equation 2:

(2) $$y=\;\frac{y_{min}\;+\;\left(y_{max}-y_{min}\right)}{1+10^{\left(\left(logIC_{50}-x\right)*slope\right)}}$$

Here, y_max_ is the smallest, and y_min_ is the highest amount of inhibition, respectively, and x represents the concentration of the inhibitor. The IC_50_ value is defined as the concentration necessary to obtain 50% inhibition.

In case of both substrate stimulations, followed by substrate inhibition after maximal stimulation was reached, the modulation (%) was plotted against the substrate concertation and data was analyzed using by two fits: First part (stimulation) was as usually fitted using Equation 1. The second part (substrate inhibition) was analyzed by Equation 3 starting at the last point of the plateau:

(3) $$y=\;\frac{y_{min}+\left(y_{min}-y_{max}\right)}{\left(1+\frac{x}{IC_{50}}\right)}$$

Here, y_max_ is the highest value of the plateau after stimulation is reached, while y_min_ is the smallest value of y after maximal stimulation was reached. The IC_50_ value is defined as the concentration necessary to obtain 50% reduction. Mathematical analysis was performed using Prism (version 8, GraphPad). All experiments were performed as at least triplicates, if not otherwise stated.

### Determination of the critical micelle concentration

CMC values were determined as described (61). Here, the fluorescence of Hoechst 33342 is measured in a concentration-dependent manner. 96-well plates suitable for fluorescence spectroscopy (Greiner Bio-One, FIA plate, black, flat bottom, medium binding) were used. Final concentration of Hoechst 33342 was set to 7 μM. CMCs of the bile acids were determined in ATPase reaction buffer (50 mM Tris-HCl, pH 7.5 (at 37°C), 100 mM NaCl) containing no FC-16, MgCl_2_, or ATP at 37°C using a Tecan M200 plate reader (Atlantic lab equipment). Bile acids were dissolved either in ddH_2_O or in 100% DMSO (as stated in “Lipid, cholesterol and bile acid preparation”) at a concentration of 100 mM. Different amounts of the stock solution were added into each well containing already the reaction buffer and Hoechst 33342. In case of hydrophobic bile acids, which were dissolved in DMSO, DMSO concentration was adapted to 1% in all wells. Emission spectra were recorded with filters set to λ_ex_ = 355 ± 10 nm, λ_em_ = 460 ± 80 nm. After background correction, fluorescence data were analyzed using Prism (version 8, GraphPad) with Equation 4:

(4) $$F=\;\frac{F_{max}*\left(c\left[BA\right]-cmc\right)}{\left(K_{0.5}-cmc\right)+\left(c\left[BA\right]-cmc\right)}$$

The measured fluorescence at each bile acid concentration (c[BA]) is represented by F, while F_max_ is the maximal fluorescence, K_0.5_ is the midpoint of the function, and cmc is the critical micelle concentration of the bile acid.

## Results

### Expression and purification of ABCB4 wild type by tandem affinity purification

**Fig. 2:**
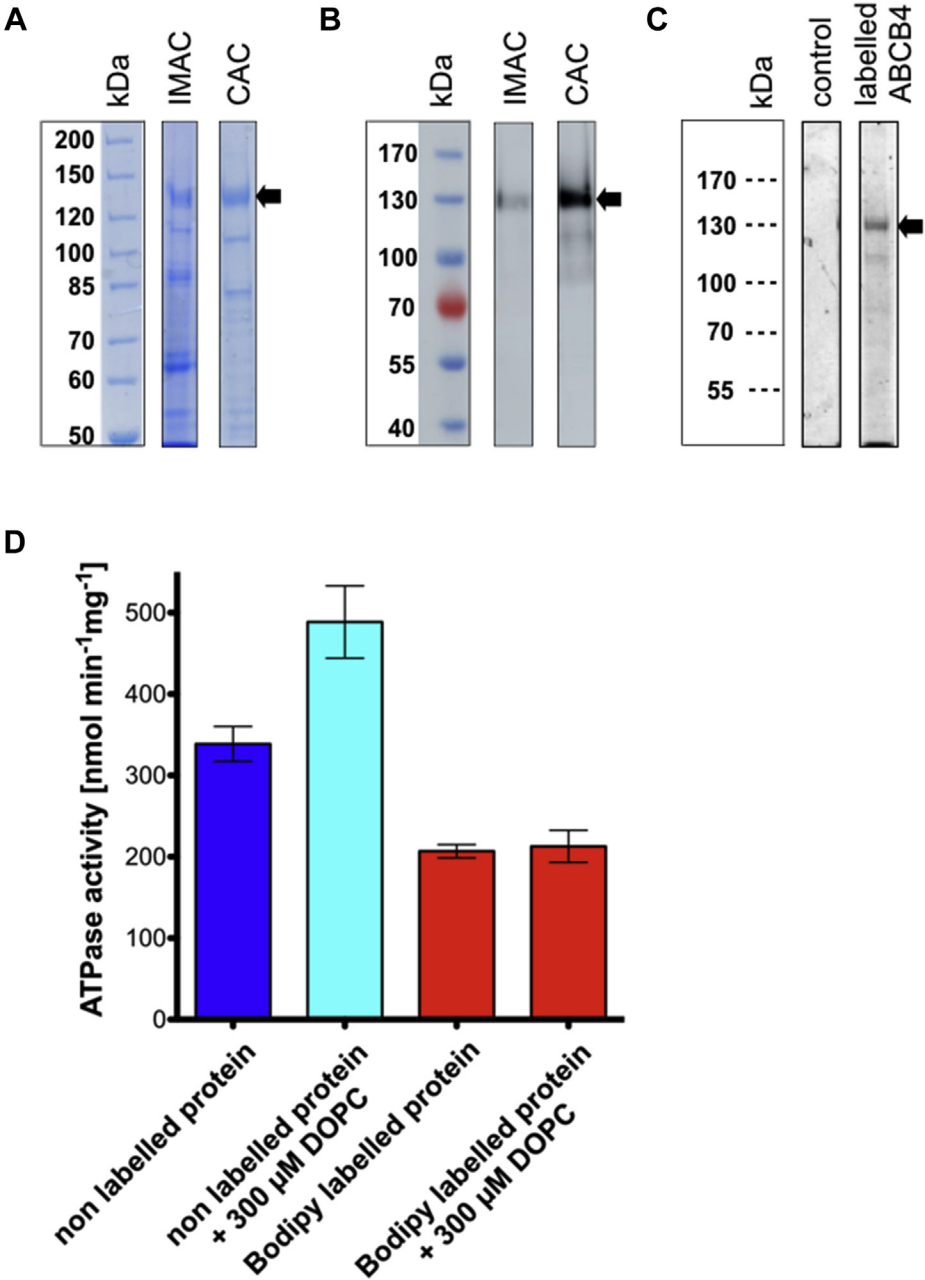
ABCB4 purification, labeling, and ATPase activity. Human *wild-type* ABCB4 purified from *P. pastoris*. ABCB4 contains C-terminally a calmodulin binding peptide and two 6xHis-tag. It was purified by tandem affinity chromatography (first an IMAC, second a calmodulin affinity chromatography (CAC)). In total, 10 μl of the elution fractions of the IMAC and CAC was mixed with 40 μl SDS-buffer and injected on the gels for analysis by colloidal Coomassie brilliant blue staining (A) and immuno-chemiluminescence using the monoclonal anti P-gp C219 antibody (B). C: Purified ABCB4 was exceeded with 10-fold molar excess of BODIPY® FL maleimide (Bodipy). In total, 10 μl of an unlabeled control and the labeled ABCB4 were mixed with 40 μl of SDS-buffer and run on an 7% SDS-gel. Bodipy emission was detected under UV excitation. D: ATP hydrolysis activity using 5 μg of protein from the CAC elution fraction (blue) and with additional 300 μM DOPC (cyan). Red bars show the ATP hydrolysis of 5 μg of Bodipy®-labeled ABCB4 with and without the addition of DOPC. Data represent the mean and SD of three biological independent experiments.

Previously, the expression of chromosomally integrated wild-type ABCB4 in *P. pastoris* was described (43, 53, 54). Fermentation led to an average yield of 1.5 kg wet cell weight. Additionally, FC-16 is suitable to solubilize ABCB4 in large quantities (53). ABCB4 was solubilized in 1% FC-16, and the supernatant was applied to a tandem affinity chromatography procedure after a sequential centrifugation step (43, 53, 54). Purification was analyzed by Colloidal Coomassie Brilliant blue–stained SDS PAGE gels (Fig. 2A) and immunoblotting using the monoclonal P-gp C219 antibody, which also recognizes ABCB4 (Fig. 2B). On average, a yield of 5–6 mg of protein out of 100 g wet cell weight was obtained with a homogeneity of approximately 80%–85%.

### ATP hydrolysis of purified ABCB4 and BODIPY® FL maleimide-labeled ABCB4

Previously, it was demonstrated that cross-linking the cysteine of the Walker A motif in ABCB1 (P-gp) with maleimide derivates resulted in inhibition of ATP hydrolysis of ABCB1 (58) and was used to study the function of ABCB1 (62, 63). Since ABCB4 also contains such a cysteine in the Walker A motif, BODIPY® FL maleimide (further referred as Bodipy) was used to inhibit specifically ABCB4. Hence, kinetic parameters of ATP hydrolysis of ABCB4 wild type, the EQ-double mutant, and Bodipy-labeled wild-type protein in the presence and absence of 300 μM DOPC, respectively, have been already determined (54). Under UV excitation, an SDS PAGE gel highlights that signal for only the labeled sample at the height of the 130 kDa marker band was observed (Fig. 2C) indicating successful labeling.

As a proof of concept, ATP hydrolysis of ABCB4 and its BODIPY-labeled form was determined in the absence and presence of DOPC (Fig. 2D), respectively. Red bars represent the ATPase activity of ABCB4-Bodipy in the absence and presence of DOPC, which was identical within experimental error (206.8 ± 8.2 nmol min^−1^ mg^−1^ without DOPC and 212.8 ± 19.7 nmol min^−1^ mg^−1^with DOPC). This indicates that the non-ABCB4-mediated ATPase activity is not influenced by the addition of DOPC. Nonlabeled protein displayed an ATPase activity of 338.8 ± 21.3 nmol min^−1^ mg^−1^ and 488.6 ± 44.5 nmol min^−1^ mg^−1^ in the absence and presence of 300 μM DOPC, respectively. These values are in good agreement with published data (54). The specific ATPase activity of ABCB4 was calculated by subtracting the activity of Bodipy-labeled ABCB4 from the activity of the nonlabeled membrane protein. Thus, ABCB4 possessed a basal activity of 132.1 ± 13.0 nmol min^−1^ mg^−1^ and was stimulated twofold in the presence of DOPC (275.8 ± 24.8 nmol min^−1^ mg^−1^). Additionally, only ABCB4 was stimulated by DOPC since the activity of the Bodipy-labeled protein remained the same in the presence or absence of DOPC.

### ATPase activity of purified ABCB4 is modulated by conjugated bile acids

**Fig. 3:**
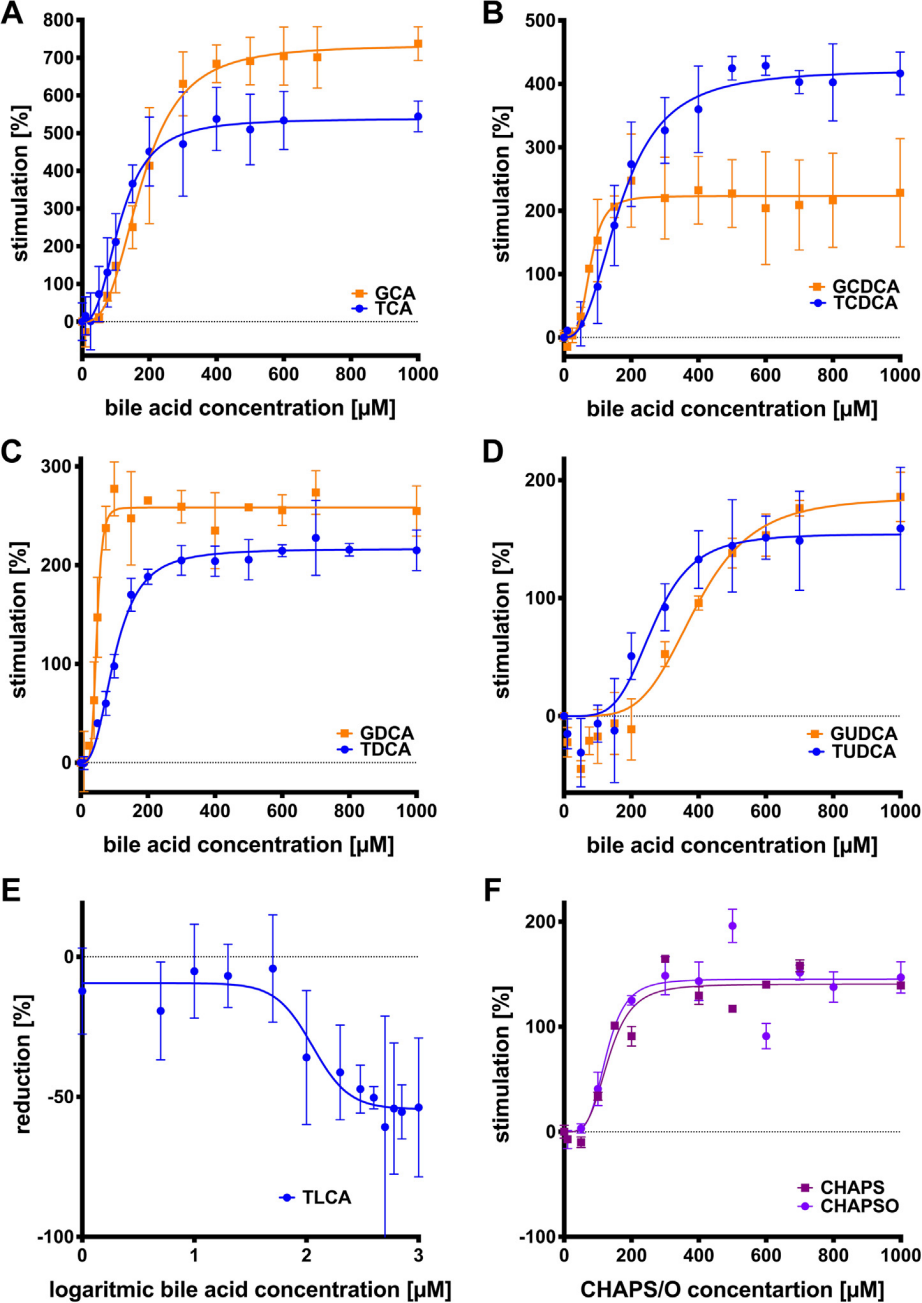
Modulation of the specific ABCB4 ATP hydrolyzing activity by the different conjugated bile acids. Modulation presented as % stimulation/% reduction based on the basal activity at 0 μM bile acid, which was set to 0%. Modulation of the ATPase activity of ABCB4 was measured in concentration-dependent manner from 0 to 1,000 μM of the glycine (orange) and taurine (blue) versions of cholic acid. Please note the different scaling of the *y* axis in the different panels. A: Chenodeoxycholic acid (B), deoxycholic acid (C), and ursodeoxycholic acid (D). For lithocholic acid (E), only the taurine-conjugated version was soluble in 5% DMSO, while glycolithocholic acid precipitated at higher concentrations (>100 μM). Since the curve of TLCA presents an IC_50_ fit, the bile acid concentration (*x*-axis) is presented as their corresponding decadic logarithmic values. Additionally, the bile-acid-derived detergents CHAPS (dark violet) and CHAPSO (bright violet) were analyzed at the same concentration as bile acids (F). A–D: Data point represent the average of three independent experiments with the errors reported as SD and data were analyzed according to an allosteric sigmoidal fit (Equation 1). E: Bars and error bars represent the mean and SD of three independent experiments and were analyzed using Equation 2. F: Data are presented as mean and SD of two independent experiments and were fitted according Equation 1.

This in vitro setup was used to address the question whether or not conjugated bile acids have an direct effect on the ATPase activity of ABCB4. We investigated both glycine and taurine-conjugated versions of human bile acids, because the majority of bile acids are conjugated with either glycine or taurine in the natural environment of ABCB4. Indeed, a modulation of ATPase activity by different bile acids was observed (Fig. 3). Since bile acids are detergents and modulate the activity of other ATPases, we again employed labeling with Bodipy® and subtracted this activity as control. No bile-acid-modulated ATPase activity of such a sample was detected. As described in Materials and Methods, conjugated bile acids were used in the range of 0–1,000 μM. However, due to batch-to-batch variations of detergent-solubilized and purified ABCB4, we decided to present all data as normalized values, in which the basal activity of ABCB4 (in the absence of PC lipids, bile acids, or cholesterol) was set to 0% and all other values are calculated as percent of stimulation of the basal activity. Thus, we determined comparable levels of stimulation and the EC_50_ for individual bile acids. Except for TLCA, all conjugated bile acids stimulated the ATPase activity of ABCB4 (Fig. 3A–D). Glycolithocholic acid precipitated at concentration higher than 100 μM and was not included in our quantitative analysis.

The highest stimulation was observed for the conjugated primary bile acids G/TCA (Fig. 3A). At concentration lower than 50 μM, these bile acids did not modulate ATP hydrolysis within experimental error. In the range of 75–200 μM for TCA (EC_50_ = 114 ± 10 μM) and 75–300 μM for GCA (EC_50_ = 177 ± 10 μM) stimulation increased before reaching the maximal stimulatory effect at approximately 400 μM. Stimulation reached 733 ± 24% for GCA and 539 ± 24% for TCA, respectively (Table 1). The primary bile acid taurochenodeoxycholic acid (TCDCA) stimulated ABCB4 activity up to 423 ± 15% (Fig. 3B), which is the third highest stimulation that was observed. The corresponding glycine-conjugated bile acid, GCDCA, reached a maximal stimulation of 223 ± 10% (Fig. 2B). Parallel to G/TCA, the data of G/TCDCA showed no effect at concentration lower than 50 μM, followed by an increase until the maximal stimulation was reached at approximately 150 μM for GCDCA and approximately 500 μM for TCDCA. This implied that the conjugated primary bile acids act in a similar manner and only the maximal stimulation was affected due to differences in the structure and/or physiochemical properties of these bile acids. The secondary bile acids G/TDCA (Fig. 3C) differed not substantially from the primary bile acids. Glycine-conjugated DCA excelled the corresponding taurine-conjugated version by approximately 40%, with maximal stimulations for G/TDCA of 259 ± 5% and 217 ± 4%, respectively (Table 1).

**Table 1:** Kinetic parameters of ATPase activity of ABCB4 in the presence of conjugated bile acid

Bile Acid	Max. Stimulation (%)	EC_50_ (μM)	Slope
GCA	733.2 ± 23.5	177.0 ± 9.9	2.9 ± 0.4
TCA	538.5 ± 24.1	114.4 ± 9.7	2.6 ± 0.5
GCDCA	223.2 ± 10.1	77.8 ± 7.5	3.9 ± 1.5
TCDCA	422.7 ± 15.1	169.2 ± 11.0	2.6 ± 0.4
GDCA	258.5 ± 4.8	47.9 ± 1.5	5.9 ± 1.2
TDCA	216.5 ± 3.9	101.8 ± 4.2	2.7 ± 0.3
GUDCA	185.3 ± 12.9	393.2 ±23.3	4.5 ± 1.0
TUDCA	154.4 ± 10.8	266.5 ± 24.2	4.5 ± 1.4
CHAPS	142.6 ± 11.5	150.9 ± 20.8	3.7 ± 2.1
CHAPSO	145.4 ± 15.3	124.9 ± 23.3	4.3 ± 2.5
Bile acid mixture	566.0 ± 17.6	430.4 ± 11.2	4.5 ± 0.4

	Max. Inhibition (%)	IC_50_ (μM)	Slope
TLCA	−54.4 ± 5.8	113.1 ± 1.4	−2.7 ± 2.1

Due to their high relevance in medicine, the conjugated versions of the bile acid UDCA were also considered. G/TUDCA are secondary bile acids in humans although the only difference from the primary G/TCDCA is the configuration of the hydroxyl moiety at position 7. A maximal stimulation of 185 ± 13% and 154 ± 11% was observed for G/TUDCA, respectively (Table 1). However, clear differences in the shape of the curves for G/TUDCA were apparent. The range, in which no stimulation occurred, was extended. The mean values at 50 μM showed a reduced activity of 30%–45%. Hence, stimulation of ABCB4 required higher concentrations of bile acid (Fig. 3D) and therefore EC_50_ values increased (Table 1). Half-maximal stimulation for G/TUDCA (EC_50_ values of 393 ± 23 μM and 267 ± 24 μM, respectively) was reached at significantly higher concentrations compared with the other bile acids. One possible rational for this behavior might be the unusual configuration of the hydroxyl moiety at position 7, which is beta in these cases. Nevertheless, all these bile acids showed the same modulation pattern. No modulation at lower concentrations followed by an increase, which finally resulted in a plateau value.

An opposite behavior was observed for TLCA. LCA makes up only ∼1.5% of the total bile in healthy humans (8) and is more hydrophobic than the other bile acids, because it lacks two hydroxyl groups compared with, for example, CA. It is used as a model compound to induce cholestasis in rat liver (64, 65, 66, 67). TLCA demonstrated inhibition of the ATPase activity of ABCB4 (Fig. 3E). Similar to the other bile acids, no effect on ABCB4 at concentrations below 50 μM was observed. At 100 μM ABCB4 ATPase activity was reduced by 36%. Maximal inhibition of approximately −55% was reached at 400 μM TLCA, which is in the range in which other bile acids had already reached v_max_. The half inhibitory concentration (IC_50_) was 113 ± 1.4 μM and therefore in comparable range to the EC_50_ values of bile acids, which stimulated ABCB4 (except for G/TUDCA).

To investigate the importance of conjugation, the two bile-acid-based detergents 3-[(3-cholamidopropyl)dimethylammonio]-1-propanesulfonate (CHAPS) and 3-[(3-cholamidopropyl)dimethylammonio]-2-hydroxy-1-propanesulfonate (CHAPSO) were tested. Both may be synthesized from CA and have a more bulky and complex conjugation than G/TCA. CHAPS and CHAPSO are presented as mean and error of duplicates (Fig. 3F). Within experimental errors, both compounds stimulated ATPase activity of ABCB4 in an identical pattern. Below a concentration of 100 μM, no stimulation was observed. Instead, slight inhibition occurred at 10 and 50 μM for CHAPS (–7.1 ± 3.6% and –9.9 ± 5.0%) and at 10 μM for CHAPSO (–7.5 ± 8.6%). At concentration higher than 50 μM, ATPase activity increased, until the maximal stimulation of 142.6 ± 11.5% and 145.4 ± 15.3% for CHAPS and CHAPSO, respectively, was reached. The plateau started at a concentration of 300 μM for both. Hence, the EC_50_ values for CHAPS and CHAPSO were in close range to each other (EC_50_(CHAPS): 150.9 ± 20.8 μM and EC_50_(CHAPSO): 124.9 ± 23.3 μM, Table 1).

### ATPase activity of purified ABCB4 is modulated by unconjugated bile acids

The half synthetic bile acid Nor-UDCA, which is often employed as a new drug for cholestatic liver diseases, remains unconjugated, because its shorter side chain inhibits coenzyme A formation (68). To compare values of unconjugated Nor-UDCA, all major bile acids were tested in their unconjugated state. This also should provide insights in the importance of conjugation for the modulation of ATPase activity of ABCB4.

**Fig. 4:**
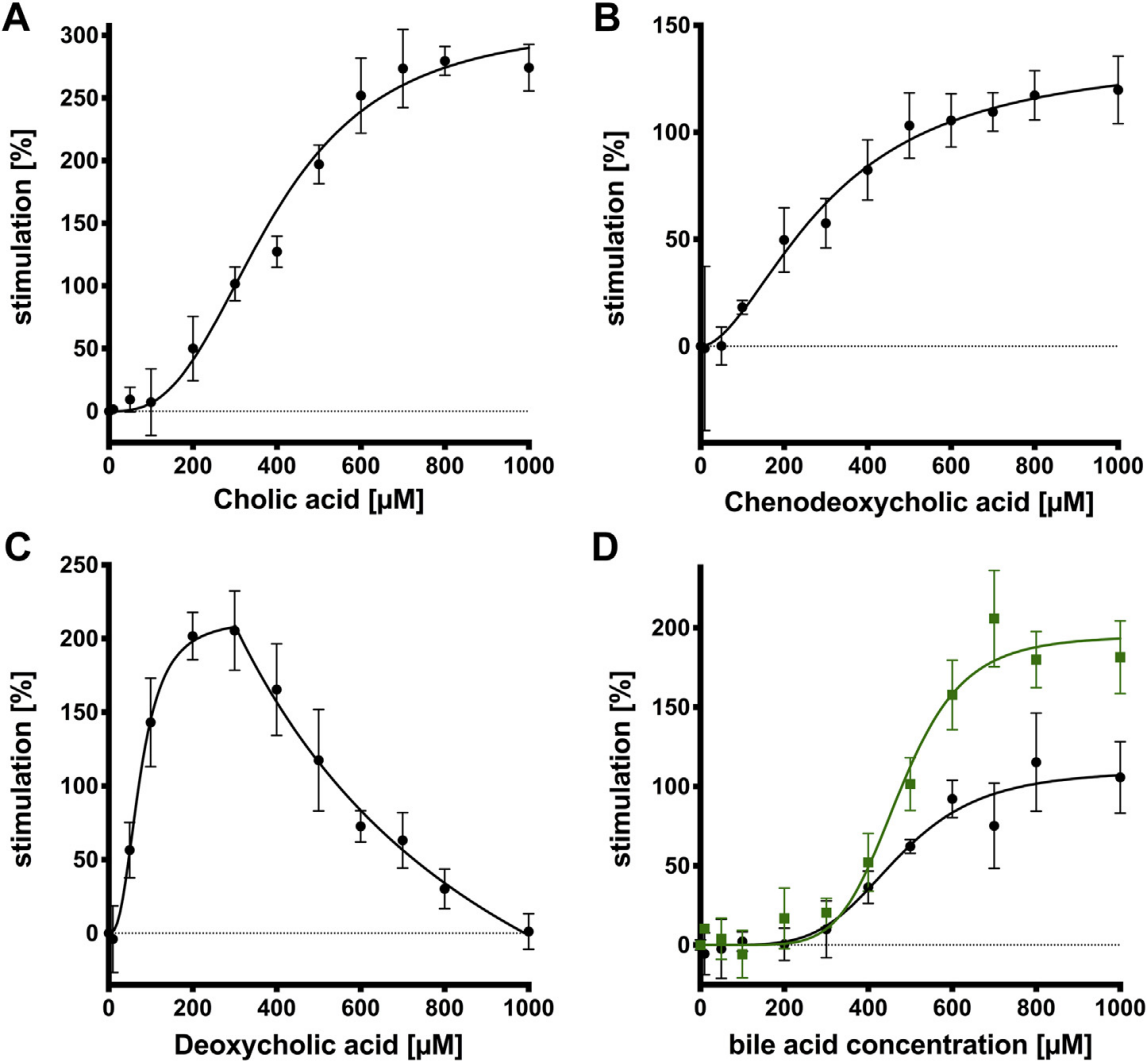
Modulation of the specific ABCB4 ATPase activity by the different unconjugated bile acids. Modulation is presented as stimulation in percentage based on the basal activity at 0 μM bile acid, which was set to 0%. Modulation of the ATPase activity of ABCB4 was measured in concentration-dependent manner from 0 to 1,000 μM the bile acid cholic acid (CA). Please note the different scaling of the *y* axis in the different panels. A: Chenodeoxycholic acid (CDCA) (B), deoxycholic acid (DCA) (C), and ursodeoxycholic acid (UDCA) as well as the side chain shortened variant Nor-UDCA (D). Lithocholic was excluded since it was not soluble under the assay conditions at higher concentrations. For DCA kind of “substrate” inhibition after maximal stimulation was measured. For a better overview in panel D, Nor-UDCA was highlighted in green. Data point represents the average of three independent experiments with the errors reported as SD. Data were analyzed according to an allosteric sigmoidal fit (Equation 1) except for C. Here, stimulation was analyzed similar to others according to an allosteric sigmoidal fit (Equation 1), while the substrate-induced reduction is fitted according to Equation 3.

The highest stimulation for the unconjugated versions was obtained from CA (Fig. 4A) with a v_max_ value of 310.5 ± 16.4% (Table 2). Similar to the conjugated versions at concentrations lower 100 μM, CA did not modulate ATPase activity within experimental errors. The half-maximal stimulation (EC_50_) was reached at a bile acid concentration of 390.5 ± 22.7 μM. The maximal stimulation was reached at approximately 700–800 μM. Similar results were observed for the other unconjugated primary bile acid CDCA (Fig. 4B). Within error no modulation occurred below 50 μM. Stimulation of the ATPase activity started at 150 μM and reached also its maximum at 700–800 μM CDCA with a maximal stimulation of 137.1 ± 16.8% and therefore is significantly lower than for CA (Table 2). The EC_50_ value of CDCA (308.4 ± 56.8 μM) is also decreased compared with CA (390.5 ± 22.7 μM). For the secondary bile acid DCA, a different modulation pattern was observed (Fig. 4C). Although no modulation within experimental errors below 50 μM occurs, the stimulation increased rapidly to a maximum of 213.6 ± 13.3% at a concentration of 300 μM. Therefore, the EC_50_ value of DCA (75.0 ± 7.3 μM) is significantly lower compared with the others. Unlike all previous bile acids, DCA has no plateau after reaching maximal stimulation. Starting at concentration of 400 μM, the ATPase activity is reduced to 0% within the errors if 1,000 μM of DCA is present. This might indicate “substrate” inhibition and is only observed for this bile acid. Next, the two medically relevant bile acids UDCA and Nor-UDCA were analyzed (Fig. 4D). Both bile acids have no modulatory effect within experimental errors up to 200 μM and share a nearly identical EC_50_ value of 470.3 ± 34.5 μM (UDCA) and 475.8 ± 18.5 μM (Nor-UDCA, Table 2). Additionally, both reach the maximal stimulation at the same concentration (700–800 μM). However, the v_max_ of Nor-UDCA exceeds the v_max_ of UDCA. More precisely, the maximal stimulation of Nor-UDCA with a value of 195.0 ± 10.8% is the highest of all unconjugated, two hydroxy bile acids (CDCA, DCA, UDCA, and Nor-UDCA) and second highest of all unconjugated bile acids, except CA. In contrast, UDCA only reaches a maximal stimulation of 110.3 ± 10.7% and therefore has the lowest stimulatory effect among the unconjugated bile acids. In summary, all unconjugated bile acids stimulate the ATPase activity of ABCB4. While CA, CDCA, UDCA, and Nor-UDCA show an overall similar curve, DCA is the first and only bile acid to show a reduction in stimulation after reaching the maximum (bell-shaped curve). One has to highlight again that stimulation by Nor-UDCA exceeds all other unconjugated bile acids, except for CA.

**Table 2:** Kinetic parameters of ATPase activity of ABCB4 in the presence of unconjugated bile acid and cholesterol

Bile Acid	Max. Stimulation (%)	EC_50_ (μM)	Slope
CA	310.5 ± 16.4	390.5 ± 22.7	2.8 ± 0.3
CDCA	137.1 ± 16.8	308.4 ± 56.8	1.8 ± 0.4
DCA	213.6 ± 13.3	75.0 ± 7.3	2.6 ± 0.6
UDCA	110.3 ± 10.7	470.3 ± 34.5	4.6 ± 1.3
Nor-UDCA	195.0 ± 10.8	475.8 ± 18.5	6.3 ± 1.3
Cholesterol	103.5 ± 5.9	0.7 ± 0.05	7.8 ± 2.2

### Modulation of ABCB4 by bile acids is correlated to their cmc

From a chemical point of view, bile acids differ only in one or two moieties (Fig. 1). All have a hydroxyl moiety at position 3 in common. The bile acid with the highest number of hydroxy moieties is CA and its derivatives. It is hydroxylated at position 7 as well as position 12. Importantly, CA showed the highest maximal stimulation regardless of conjugation or not. In contrast, LCA and its derivates lack both of these hydroxy moieties and TLCA was the only bile acid that inhibited basal activity of ABCB4 in our setup (Fig. 3E). All other bile acids possess a hydroxy moiety at position 7 or 12. Furthermore, their maximal stimulation was between the corresponding CA and LCA derivates. Additionally, the kinetics of G/TUDCA bile acids with a hydroxy group at position 7 in the β-conformation revealed a shift in the EC_50_ values (Table 1). Therefore, ABCB4 might recognize the cholesterol backbone and especially the hydroxy groups at position 7 and 12.

To analyze and compare all bile acids, we determined the cmc of each bile acid under the conditions of our assay. We used an assay, which relied on the increase of the fluorescence of Hoechst 33342 in the hydrophobic environment of a micelle (61). Although cmc values for many bile acids have been reported (69, 70, 71, 72, 73, 74), cmc values are sensitive toward parameters such as temperature, pH, and/or ionic strength. For every bile acid used in the ATPase activity assay, a cmc was determined and summarized in Table 3. The primary bile acids G/TCA have the highest cmc values (4.1 and 3.5 mM), while for unconjugated CA a cmc of 2.3 mM was determined. Conjugated versions of CDCA and DCA exhibit a cmc in the range from 1.7 mM to 2.6 mM. The cmc of unconjugated CDCA and DCA is lower than the corresponding conjugated versions but nearly identical (1.5 mM for CDCA and 1.4 mM for DCA, respectively). Interestingly, G/TUDCA and unconjugated UDCA displayed cmc in a narrow range. Among them GUDCA has the highest cmc (1.6 mM), second is TUDCA (1.5 mM), and hence, UDCA (1.3 mM) has the lowest of these three bile acids. Here, conjugation has less to no impact on hydrophobicity and cmc. Shortening the side chain of UDCA therefore resulted in an increase of the cmc. Nor-UDCA possessed a cmc of 1.8 mM. In literature, UDCA and its versions are considered to have one of the highest cmc (10, 75). However, we clearly observed an increase in fluorescence emission already in the range of 1.3–1.8 mM for our UDCA versions. The harmful bile acid TLCA possess a cmc of 0.14 mM and is therefore the only bile acid in our study with a cmc significantly below 1 mM. The determined cmc values of the bile acids demonstrate that monomeric bile acids modulate the ATPase activity of ABCB4 as maximal stimulation was observed below a concentration of 1 mM bile acids, except for TLCA. Hence, the stimulatory effects are based on an interaction of ABCB4 with monomeric bile acids, not with the micelles. Additionally, the different maximal stimulations are reached due to the properties of bile acids and not because the free bile acid concentration is limited by the formation of micelles.

**Table 3:** Critical micelle concentration of bile acids under assay conditions

Bile Acid Conjugation	CA (mM)	CDCA (mM)	DCA (mM)	UDCA (mM)	Nor-UDCA (mM)	LCA (mM)
Glycine	4.1 ± 0.1	2.6 ± 0.1	2.0 ± 0.1	1.6 ± 0.1	---	Precipitate^a^
Taurine	3.5 ± 0.1	1.7 ± 0.1	1.7 ± 0.1	1.5 ± 0.2	---	0.14 ± 0.03
Unconjugated	2.3 ± 0.2	1.5 ± 0.1	1.4 ± 0.1	1.3 ± 0.1	1.8 ± 0.1	Precipitate^a^

Presented are the calculated values and the error of the mean derived from the fitting procedure. Data were evaluated using Equation 4. For Nor-UDCA, only the unconjugated version was considered, since it is not conjugated in hepatocytes.

a G/LCA precipitated at concentrations higher than 100 μM in our setup.

**Fig. 5:**
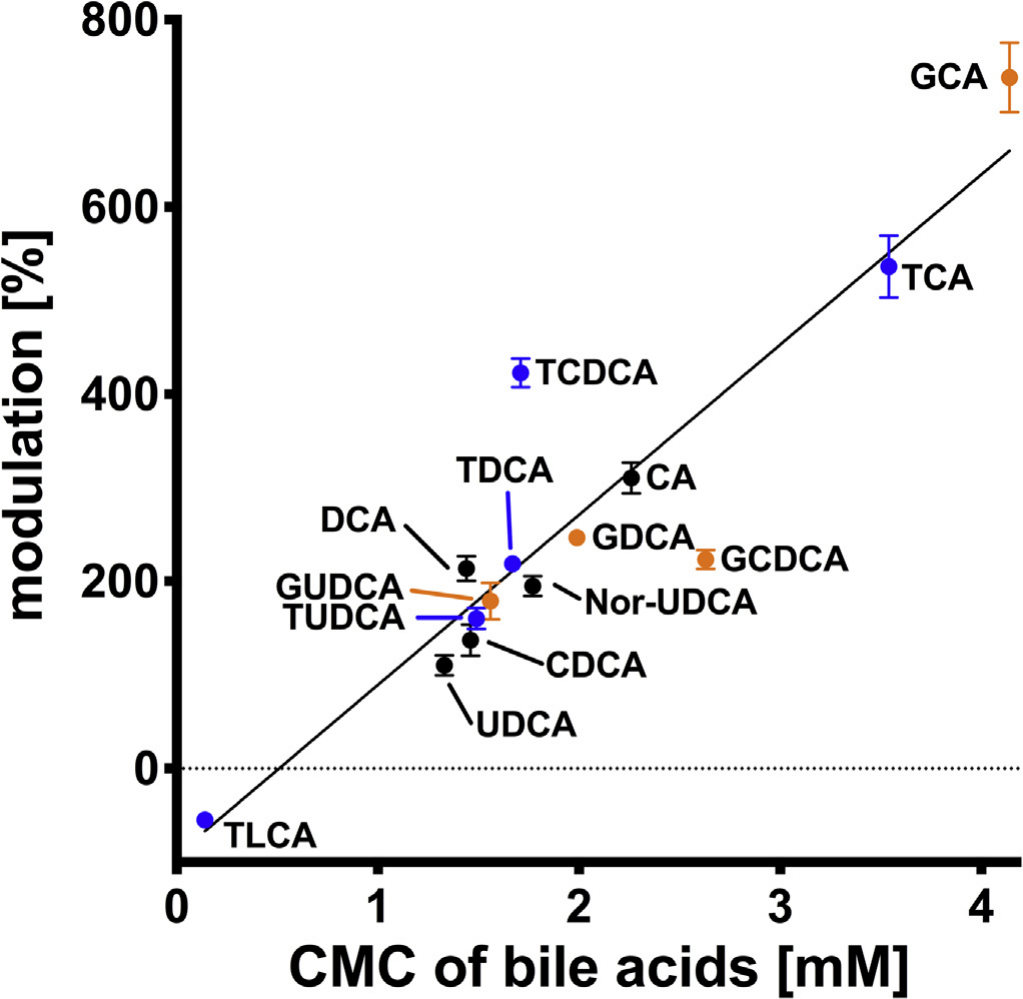
Dependence of bile acid modulation of ABCB4 and cmc. Glycine-conjugated bile acids are shown in orange, taurine-conjugated bile acids are colored blue, and the unconjugated versions are in black. Linear regression revealed a slope of 181.8 ± 12.9 and a correlation coefficient *r*^2^ of 0.83. Data point represents the v_max_ values and errors of individual bile acid kinetics (Figs. 3 and 4, Tables 1 and 2). CMC values were taken from Table 3.

Plotting the maximal stimulation against the cmc values revealed a nearly linear relation with a correlation coefficient *r*^2^ of 0.83 (Fig. 5). The highest stimulatory effect was observed for GCA, which also has the highest cmc value (4.1 mM). In contrast, TLCA was the only bile acid that reduced the ATPase activity of ABCB4 and exhibits the lowest cmc value (0.14 mM). Hence, formation of micelles already starts at 140 μM, and we cannot distinguish whether the observed reduction is due to TLC itself or because of micelle formation. In summary, we determined cmc values under the same conditions as in the ATPase activity assay for each bile acid in this study. Further and more important, a linear correlation between maximal stimulation and cmc values was observed.

### ABCB4 accepts more than one bile acid

**Fig. 6:**
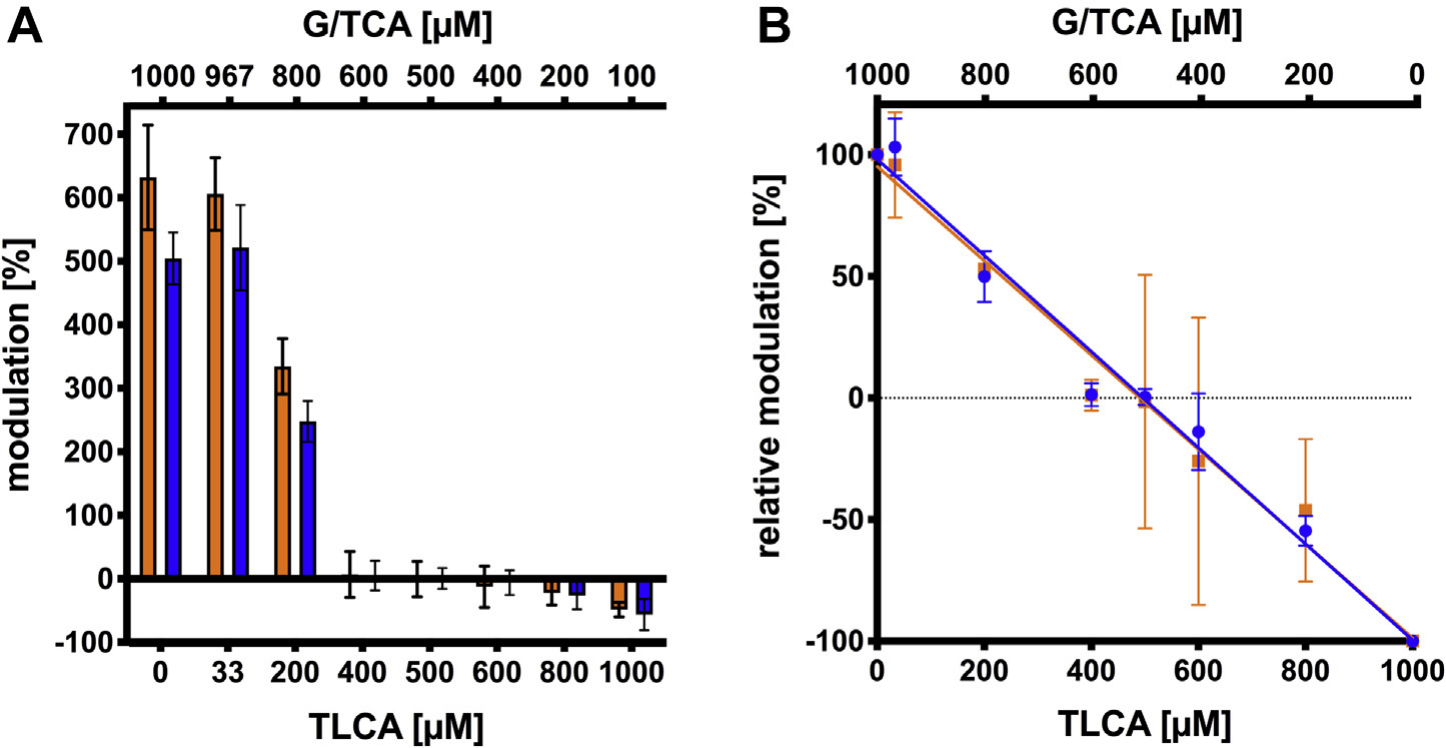
Modulation of the ATP hydrolysis of ABCB4 by different ratios of mixtures of GCA and TLCA (orange) or TCA and TLCA (blue). Starting at 1,000 μM the concertation of the primary bile acid was decreased in steps of 200 μM and the concentration of TLCA was increased in parallel. Additional mixtures of 967 μM G/TCA and 33 μM of TLCA and 500 μM of both bile acids were tested. All reactions contained a total concentration of 1,000 μM of bile acids and 5% DMSO. In (A) the modulation of the different mixtures is presented as stimulation or inhibition in relation to ABCB4 basal activity, which was set to 0%. In (B) the relative modulation is displayed, in which the maximal stimulation was set to 100% and maximal inhibition was set to –100%, respectively. The graph demonstrates the linear correlation between the two bile acids. A and B: Bars and error bars represent the mean and SD of three independent experiments. B: Data were analyzed by a linear fit.

ABCB4 is clearly modulated by monomeric, unconjugated and conjugated bile acids. However, the question arises whether the observed effects are additive or whether ABCB4 has a preference for certain bile acids. To investigate this, we analyzed mixtures of TLCA with G/TCA. G/TCA concentrations were decreased in 200 μM steps starting at 1,000 μM, while TLCA concentration was increased in the inverse manner. Hence, the final bile acid concentration was always kept constant at 1,000 μM. Additionally, two conditions were tested. First, a setup with 967 μM of G/TCA and 33 μM of TLC was analyzed, this equals to a 30:1 ratio and matches approximately the in vivo human CA:LCA ratio (8, 76). Second a 50:50% mixture of both bile acids was included (Fig. 6A). In the case of only GCA and TCA (1,000 μM), the maximal stimulation was slightly reduced compared with Fig. 3A. This might be due to the presence of 5% DMSO, which was not present in the kinetic measurements (Fig. 3A), but is necessary for keeping TLCA in solution in this setup. In both assays (GCA + TLCA and TCA + TLCA, respectively), pure TLCA showed an inhibition of approximately 50%, which is in line with the previously observed reduction (Fig. 3E). Mixing the primary bile acids with TLCA at a ratio of 30:1 had no effect on the modulation of ABCB4. However, at 800 μM G/TCA and 200 μM TLCA, the stimulatory effect already decreased to approximately half. Furthermore, at a ratio of 3:2 (600 μM G/TCA and 400 μM TLCA) values were at the level of basal ATPase activity of ABCB4. The same holds true for the inverse ratio (400 μM G/TCA and 600 μM TLCA) as well as the 50:50 mixture. Increasing the amount of TLCA resulted in a decrease of the ATPase activity.

One has to consider that TLCA is present in micelles, while G/TCA exist as monomeric bile acids. Thus, three questions arose: (1) how do both bile acids interact; (2) if and how much of the primary bile acids were integrated into TLCA micelles; and therefore (3) what is the true concentration of G/TCA in the mixtures? With our assay, we cannot answer these questions, but after normalization of the data, a linear correlation was observed (Fig. 6B). This demonstrates that ABCB4 does not distinguish between bile acids. In case of higher G/TCA concentrations, the ATPase activity of ABCB4 was stimulated, while higher TLCA concentrations decreased the ATPase activity.

### Modulation of the ATPase activity of ABCB4 by cholesterol

**Fig. 7:**
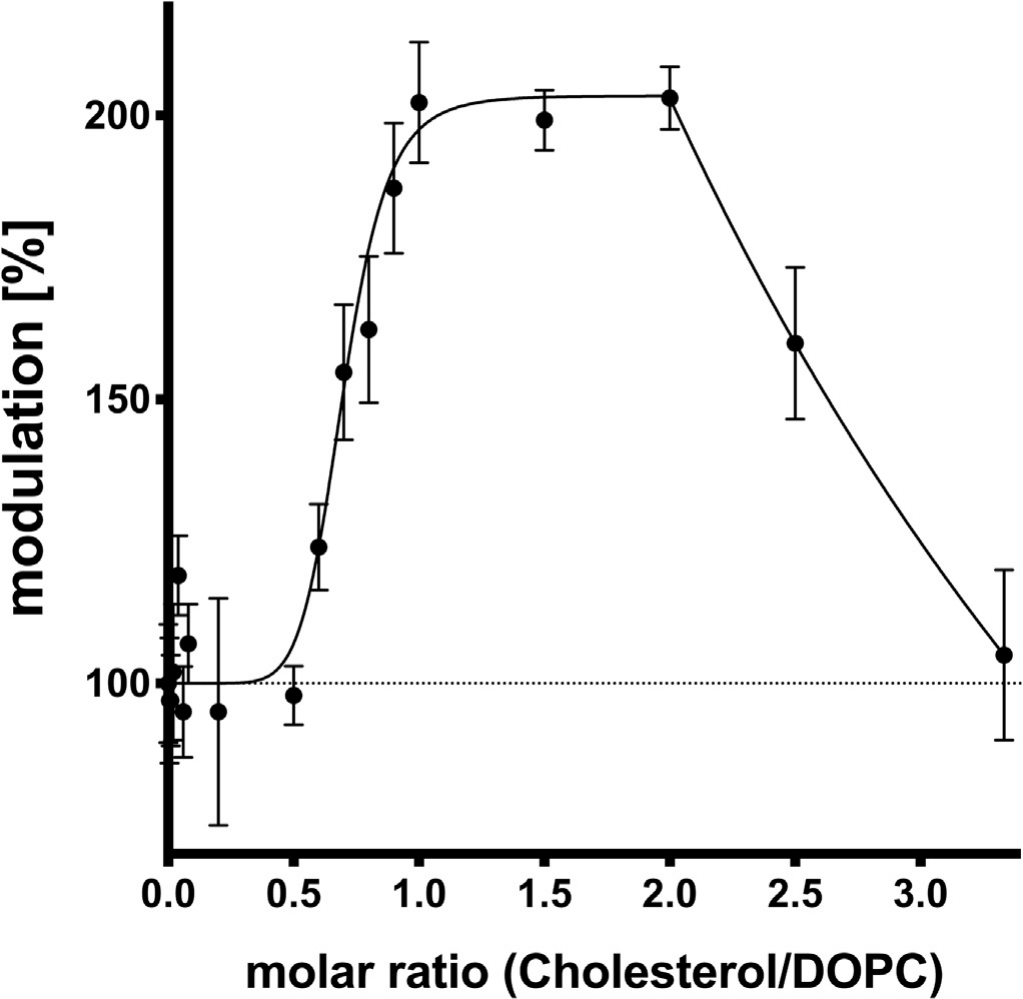
Modulation of the ATPase activity of ABCB4 by increasing portions of cholesterol in the background of DOPC. Cholesterol was mixed with DOPC (300 μM) at different ratios. Cholesterol concentration ranging from 0 to 1,000 μM resulted in molar ratios of 0–3.3 (cholesterol to DOPC). The ATPase activity at a molar ratio of 0 (only 300 μM DOPC) was set to 100% (maximal stimulation by PC lipids). Data point represents the average of three independent experiments with the errors reported as SD. Data were analyzed according to an allosteric sigmoidal fit (Equation 1) as well as Equation 3 for substrate induced reduction.

Cholesterol is abundant in every mammalian membrane and the substrate of a transporter of the bile triumvirate, ABCG5/G8. Therefore, one may assume a modulatory effect on ABCB4 similar to the one described for ABCB11 (47). Hence, this in vitro setup was used to address this hypothesis. However, a major problem of cholesterol is its solubility. To overcome this problem, chloroform is often used as a solvent, which is not compatible with our assay. Hence, cholesterol was mixed with DOPC in different ratios. In the ATPase assay, always a final concentration of 300 μM DOPC was present, mixed with cholesterol ranging from 0 to 1,000 μM resulting in molar ratios of 0–3.3 (cholesterol to DOPC). The DOPC concentration of 300 μM was chosen to ensure maximal stimulation by the PC lipid (54). Therefore, the ATPase activity at a molar ratio of 0 represents stimulation by DOPC only, which was set to 100% (maximal stimulation by PC-lipids). Here, an increase in the ATPase activity is caused by cholesterol in addition to the maximal stimulation by the natural substrate. Actually, a similar behavior as for bile acid modulation was observed. Cholesterol at molar ratios (cholesterol over DOPC) higher than 0.5 enhanced ABCB4 activity in addition to the stimulation by DOPC (Fig. 7). Below this ratio, ATPase activity remained close to DOPC only activities (100%), except for 0.04, which showed a mean value of 119% stimulation. Due to the error in the measurements, we consider this value as no stimulation. Nevertheless, for ratios higher than 0.5 (equals 150 μM cholesterol over 300 μM DOPC), stimulation up to 203.5 ± 5.9% of basal ATPase activity was observed. Since DOPC stimulation was set to 100%, it demonstrates that cholesterol doubles the ATPase activity of ABCB4 in the presence of DOPC (Table 2). This effect is limited to ratios of 1–2 (cholesterol to DOPC). A further increase of the cholesterol content resulted in a gradual decrease of activity reaching 100% within experimental error, which equals the DOPC stimulation. Nevertheless, we could demonstrate that cholesterol in the environment of an actual substrate can increase the already stimulated ATPase activity of ABCB4.

### ABCB4 is modulated in the presence of DOPC

**Fig. 8:**
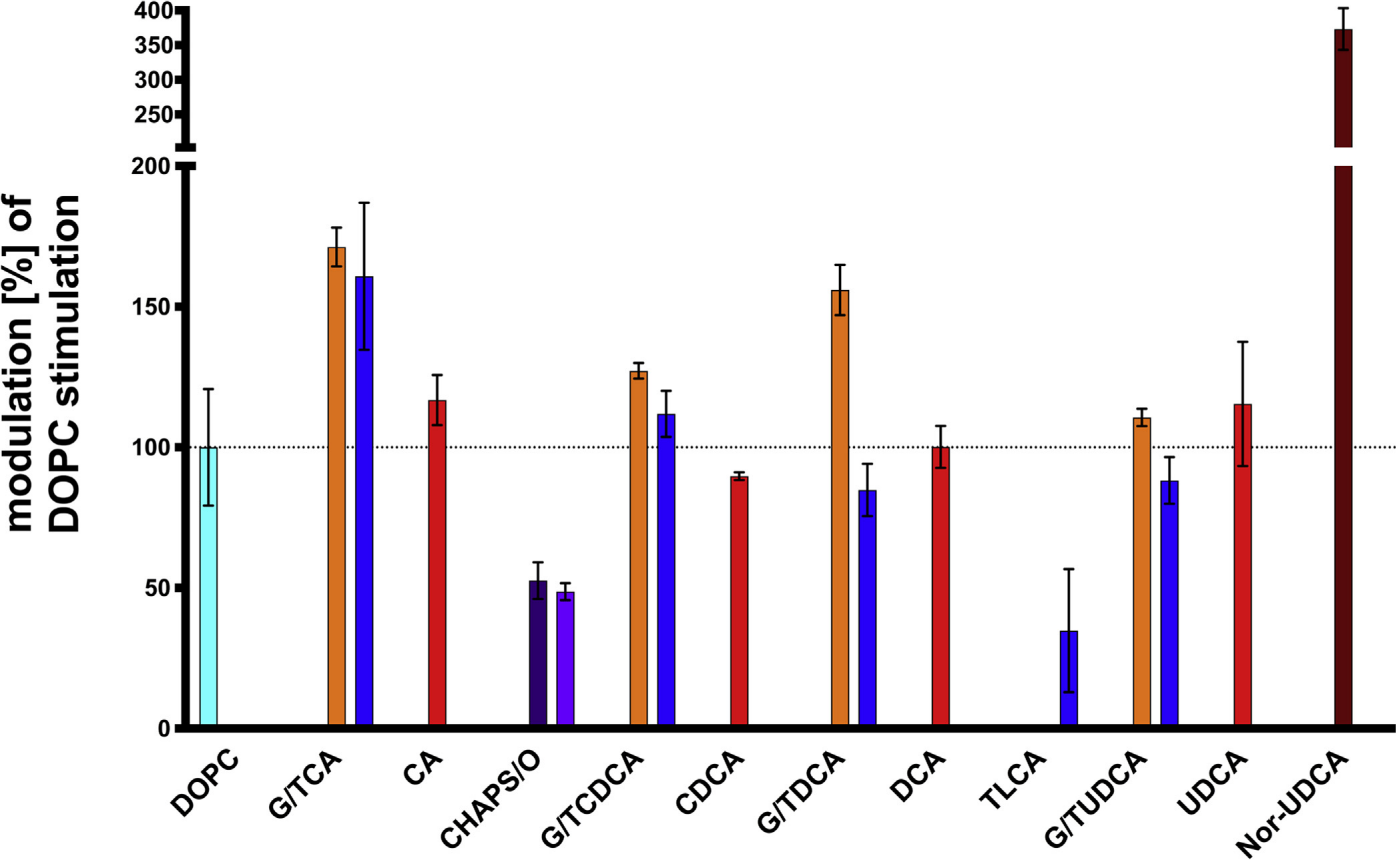
Modulation of ATP hydrolysis of ABCB4 by the different bile acids in the presence of the naturally substrate DOPC. In total, 300 μM DOPC (cyan) stimulated the ATPase activity of ABCB4 and was set to 100%. 1,000 μM of each bile acid was mixed with 300 μM DOPC and ABCB4. Glycine-conjugated variants are colored in orange, taurine-conjugated are pictured in blue, and unconjugated bile acids are shown in red. Additionally, the effect of 1,000 μM CHAPS/CHAPSO (dark/ bright violet) and Nor-UDCA (brown) was analyzed. Concentration was chosen based on the v_max_ values (Figs. 3, 4 for bile acids and Kluth *et al.* (54) for DOPC). Discontinuous ordinate was chosen for a better overview of bars below 200%. Bars and error bars represent the mean and SD of three independent experiments.

It is now commonly accepted that ABCB4 only flops lipids of the PC family from the inner to the outer leaflet of the canalicular membrane of hepatocytes (2, 40). This study demonstrated that bile acids modulate the ATPase activity of ABCB4. One has to stress that this does not suggest that bile acids are a new family of substrates. To address this question, we analyzed the modulation of ATPase activity of ABCB4 in the presence of DOPC, bile acids, and cholesterol. First, DOPC and bile acids were used at concentrations at which both substances showed maximal modulation, but below the cmc of the bile acid (except for TLCA). Data is presented as relative modulation compared with DOPC alone, which was set to 100% (Fig. 8, cyan). Both glycine (orange) and taurine (blue) conjugated versions as well as the unconjugated versions (red) of all bile acids were analyzed. Further CHAPS/O (dark/bright violet) and Nor-UDCA (brown) were tested.

GCA, TCA and unconjugated CA increased the DOPC stimulated ATPase activity of ABCB4. GCA increased ATP hydrolysis to 171.2 ± 6.9%, while maximal stimulation was observed with 160.8 ± 26.1% for TCA and at 116.8 ± 8.9% for CA. In contrast, the two structurally related detergents CHAPS and CHAPSO decreased DOPC stimulation of ABCB4 to 52.6 ± 6.5% and 48.7 ± 3.0%, respectively. For the other primary bile acid, CDCA, a slight decrease to 89.7 ± 1.4% was measured, while the conjugated versions showed an additional stimulation to 127.2 ± 2.8% and 111.9 ± 8.2% for G/TCDCA, respectively. Also, for the glycine-conjugated version of the secondary bile acid GDCA, an increase in ATPase activity for DOPC-stimulated ABCB4 was observed at 155.9 ± 8.9%. In the case of GDCA, the taurine-conjugated version TDCA lowered the ATPase activity (84.8 ± 9.3%), while unconjugated DCA had no effect (100.1 ± 7.5%). The hydrophobic bile acid TLCA decreased DOPC stimulation by approximately 65% to a minimum of 34.8 ± 21.9%, which is still above the basal ATPase activity of ABCB4. For G/TUDCA and unconjugated UDCA, no further modulation could be measured within the errors. Relative ATPase activities were observed at 110.6 ± 3.1% and 88.2 ± 8.3% for G- and TUDCA, respectively. For unconjugated UDCA, a value of 115.4 ± 22.1% was measured. In contrast, Nor-UDCA bearing a shorter side chain revealed a significant stimulation of ATPase activity of ABCB4 in the presence of 300 μM DOPC to maximum of 373.1 ± 30.0%. This is the highest ATPase activity observed within this setup and reveals the potential of Nor-UDCA.

**Fig. 9:**
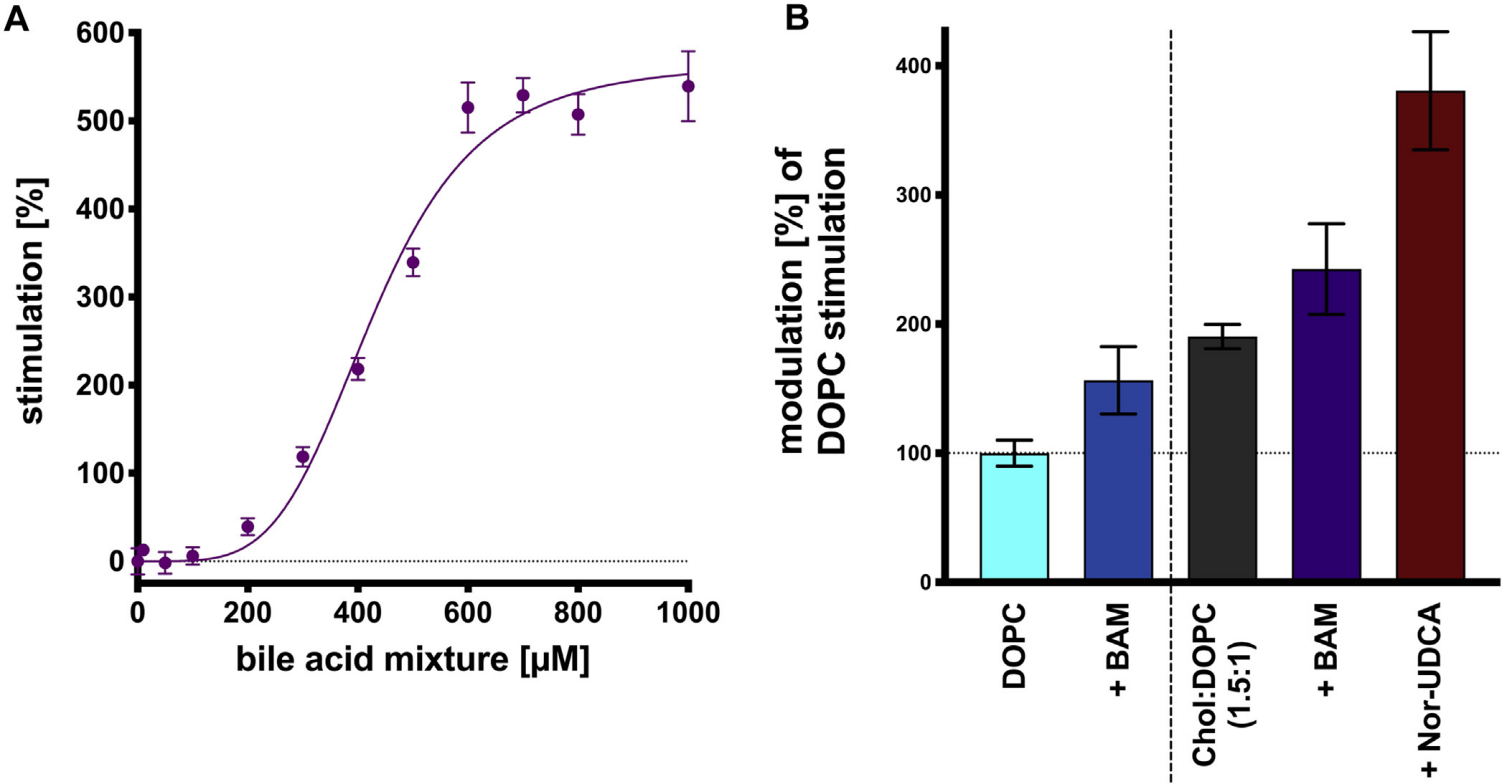
Stimulation of ABCB4 ATPase activity by a bile acid mixture (A) and stimulation of ABCB4 ATPase activity in the presence of DOPC and the bile acid mixture, cholesterol, and Nor-UDCA (B). A: The bile acid mixture (BAM) consists of GCDCA, TCDCA, GCA, TCA, GDCA, and TDCA with a ratio of 21:19:24:16:13:7 as described in literature (48, 77, 78). The BAM was tested in range from 0 to 1,000 μM similar to individual conjugated bile acids (Fig. 3). Data were analyzed according to an allosteric sigmoidal fit (Equation 1). B: Stimulation of the ATPase activity of ABCB4 by DOPC at a concentration of 300 μM (cyan bar) was set to 100% substrate stimulation (54). In all further approaches DOPC concentration was always 300 μM. Modulation of DOPC stimulated ATPase activity by the BAM is presented by the dark blue bar. Next cholesterol was added in a 1.5 M excess to DOPC (dark gray bar), so that maximal stimulation by cholesterol was achieved (Fig. 7), before the BAM (dark violet bar) or Nor-UDCA (brown bar) was added. A and B: Consider different *y*-axes. Bars and error bars represent the mean and SD of three independent experiments.

In this study, the effects of monomeric bile acids as well as monomeric bile acids in the context of DOPC and cholesterol in DOPC environment on the ATPase activity of ABCB4 were analyzed. Thus, we tried to combine these conditions. Therefore, a bile acid mixture (BAM) of GCDCA, TCDCA, GCA, TCA, GDCA, and TDCA (21:19:24:16:13:7) as described in literature was chosen (48, 77, 78). The effect of the BAM on the ATPase activity of ABCB4 was examined in a range from 0 to 1,000 μM similar to individual bile acids (Fig. 9A). For a second approach, the concentration of the BAM with highest stimulation (1,000 μM) on ABCB4 was used to investigate the effect of the BAM on ABCB4 in the presence of DOPC and cholesterol mixed with DOPC (Fig. 9B). DOPC concentration (300 μM) and cholesterol to DOPC ratio (1.5:1) were chosen based on the v_max_ values of their individual kinetics (Fig. 7 for cholesterol and Kluth *et al.* (54) for DOPC). The bile acid mixture increased ABCB4 ATPase activity in all approaches. For comparison basal ABCB4 ATPase activity was set to 0% similar to the kinetics of individual bile acids. Adding BAM resulted in a v_max_ of 566.0 ± 17.6% (Fig. 9A, Table 1), which is in good agreement with the individual kinetics of the bile acids (Fig. 3). However, in comparison to the individual conjugated bile acids present in the BAM, v_max_ is not reached until 600–700 μM. Furthermore, the EC_50_ value of BAM is significantly increased to a concentration of 430.4 ± 11.2 μM (Table 1). For the second approach, DOPC (300 μM) stimulation was set to 100% (Fig. 9B, cyan bar) similar to Fig. 8. Adding BAM to DOPC (dark blue bar) increased the ATPase activity to a maximum of 156.4 ± 26.1%. To investigate the effect of the bile acid mixture in the presence of cholesterol, first only cholesterol and DOPC in a ratio of 1.5:1 were measured (Fig. 9B, dark gray bar). Stimulation by cholesterol (190.4 ± 9.4%) is in good agreement with the v_max_ value (203.5 ± 5.9% ) of the DOPC cholesterol kinetic (Fig. 7). Hence, BAM was added to the DOPC cholesterol mixture (Fig. 9B, dark violet bar), which increased the ATPase activity further to 242.6 ± 35.1%. Since Nor-UDCA demonstrated the highest effect on ABCB4 ATPase activity in a DOPC environment, the bile acid mixture was exchanged to Nor-UDCA in the latest approach (Fig. 9B, brown bar). With a maximum of 380.7 ± 45.6%, Nor-UDCA exceeded the bile acid mixture by ∼250%. Nor-UDCA, on the other hand, was unaffected by cholesterol since stimulation in only DOPC (373.1 ± 30.0) and cholesterol:DOPC environment (380.7 ± 45.6%) was equal within the experimental error. However, the results in Figs. 8 and 9 demonstrated that maximal DOPC stimulation does not reflect the maximal stimulation of ATPase activity of ABCB4 that was observed in this study. Adding cholesterol and bile acids (independent or as mixture) increased the ATPase activity of ABCB4 beyond the DOPC value. Therefore, we conclude that bile acids are not substrates, but act as enhancers or modulators of the ATPase activity of ABCB4.

## Discussion

### ABCB4 is modulated by bile acids

The aim of this work was to answer the question if bile acids interact with ABCB4. This question derives from data demonstrating that PC lipid concentrations rises, when conjugated bile acid was added to the medium of cells or vesicles that contained ABCB4 (19, 57, 58, 59). The increased amount of PC lipids could be due to interaction with PC lipids, ABCB4, or both. For the first time, it was shown in this study that monomeric bile acids directly modulate ABCB4 (Figs. 3, 4).

We chose an in vitro setup (53), in which ABCB4 is detergent-solubilized and the composition and concentration of the compounds under investigation can be regulated under defined conditions. By tandem affinity purification, a yield of 5–6 mg of ABCB4 (Fig. 2A, B) was reached (54). Due to Bodipy labeling, it was possible to measure specific ATPase activity of ABCB4 in a background that likely contained other ATPases (Fig. 2D) (54). Although, an ATPase activity assay is not directly measuring substrate translocation, it is now generally accepted that substrates increase the protein's ATPase activity, since the NBDs change conformation and are temporally in close proximity (79).

This provided a starting point to measure ATP hydrolysis of ABCB4 in the presence of different bile acids (Figs. 3, 4). In humans, a number of bile acids are present. They vary in the number of hydroxy groups (one to three), conjugations (Fig. 1), and physical properties such as hydrophobicity. This diversity results in different cmc values for each bile acid. But the cmc of bile acids, like any other detergent, is also depended on the chemical and physical properties of the solution. Therefore, we determined the cmc of each bile acid used in this study under the conditions of the ATPase assay (Table 3). The bile acids with three hydroxy moieties G/T/CA exhibited the highest cmc (2.3–4.1 mM) and for G/TCA cmc values were in good agreement with published values (73, 74), although temperature and ionic strength were different. With decreasing numbers of hydroxy moieties, the average cmc decreased. For example, bile acids with two moieties exhibited cmc values in a range of 1.3–2.6 mM, while the bile acid with only one hydroxy moiety (TLC) displayed a cmc of 0.14 mM. Compared with literature values, our TCDCA and TDCA values are in good agreement, but cmc values of GCDCA and GDCA were slightly increased (74). In contrast, our value for TLCA is lower than that reported in a study by Hofmann and Roda (10). We could also observe that conjugation of bile acids increased the cmc for CA, CDCA, and DCA. To be more precise, the glycine-conjugated version of these bile acids exhibited always the highest cmc. All conjugated versions possessed higher cmc values than their unconjugated analogues. From a chemical point of view, this makes sense, since conjugations increase the hydrophilicity of the bile acids and as a result their cmc values. In nature conjugation of bile acids is performed to increase their water solubility (9). Regarding the importance of the position of the hydroxy moiety within the back bone and its stereochemistry, no conclusion can be drawn. While for GCDCA and GDCA, there is a difference in the cmc values between hydroxylation at position 7 (GCDCA, 2.6 mM) and position 12 (GDCA, 2.0 mM), for TCDCA (1.7 mM), TDCA (1.7 mM), CDCA (1.5 mM), and DCA (1.4 mM), no differences regarding the influence of the position of the hydroxy moiety was observed. Switching the hydroxy moiety at position 7 from the α-position (G/T/DCA) to the ß-position (G/T/UDCA) resulted in a decrease of the measured cmc for the conjugated versions (Table 3). However, shortening the side chain of UDCA by one methylene moiety (Nor-UDCA) significantly increased the cmc to 1.8 mM, which is the second highest cmc for all unconjugated bile acids.

To the best of our knowledge, the bile acid concentrations in human hepatocytes are not known. Bile concentrations in the gallbladder of healthy persons are in the range of approximately 100–150 mM (8). But gallbladder bile is highly concentrated. Therefore, we tested the effect of all bile acids on the basal ATPase activity of ABCB4 in a range of 0–1,000 μM. Indeed, all bile acids showed a modulation of the basal ATPase activity. All tri- and dihydroxy bile acids as well as the bile-acid-derived detergents CHAPS and CHAPSO demonstrated stimulation of basal ATPase activity in a concentration-dependent manner (Figs. 3, 4). Concentrations required for maximal stimulations ranged from 200 to 800 μM. More importantly, however, the maximal stimulation was always observed below their corresponding cmc values. Therefore, stimulation derives from monomeric bile acids. Furthermore, the data revealed that except for G/TCDCA, the glycine-conjugated bile acid exceeded the taurine conjugation. Interestingly, glycine conjugation is the predominant form in human bile acid. The question arises whether mice ABCB4 has an inverted preference, since taurine is the mayor conjugation in rodents. In contrast, studies investigating the effect of bile acids on lipid release in the presence of ABCB4 demonstrated that taurine-conjugated bile acids exceeded glycine-conjugated versions (76, 80, 81). However, modulation of ABCB4 and lipid extraction by bile acids are two different events that have to be considered separately. Unconjugated versions demonstrated the least maximal stimulation within the same group of bile acids. One interesting exception is Nor-UDCA, which exhibited the highest v_max_ of all UDCA variants and the second highest of all unconjugated bile acids indicating a different effect on ABCB4. All four UDCA versions had in common that their EC_50_ values are significant higher compared with the others. This is most likely due to the fact that their hydroxy moiety at position 7 is in ß-position compared with all other bile acids. In summary, monomeric tri- and dihydroxy bile acids stimulated ATPase activity of human ABCB4 in a concentration-dependent manner. Glycine-conjugated bile acids reached the highest maximal stimulation within the same group of bile acids, except for CDCA, where taurine conjugation (TCDCA) exceeded glycine conjugation (GCDCA). Additionally, higher concentration of UDCA versions was required to observe stimulation. Shortening the side chain of UDCA by one carbon atom (Nor-UDCA) revealed an increase of approximately 85% of ATPase activity.

The only exception was TLCA, a bile acid with only a hydroxy moiety at position 3. TLCA clearly decreased the activity of ABCB4. However, TLCA is also the only bile acid with a cmc significant smaller than 1 mM and therefore has to be considered separately. Up to 50 μM no effect was detected within experimental error. Approximately at the cmc, the half inhibitory concentration (IC_50_) was reached (Table 3) implying a reduction of the basal ATPase activity by monomeric TLCA (from ∼50 to 140 μM) as well as micelles (∼140–500 μM). However, after the basal activity is reduced to 50%, no further reduction was observed. This indicates that TLCA may not be able to fully inhibit ABCB4. This is in a good agreement with the fact that TLCA triggers cholestasis in rats (64, 65, 66, 67).

Interestingly plotting the maximal stimulation against the cmc values revealed a linear correlation (*r*^2^= 0.83). This correlation shows that ABCB4 recognizes bile acids by their physical properties. From a point of evolution, the most common bile acid in humans is GCA (8, 76), which also possessed the highest cmc and degree of stimulation. In contrast, UDCA and LCA represent the smallest part of the human bile acid pool (76) and displayed the lowest level of stimulation or even a reduction of basal ATPase activity. However, we could clearly demonstrate that monomeric bile acids modulate ATPase activity in dependence of their cmc. Due to the limitations of the assay, it still remains unclear how bile acids modulate ATP hydrolysis of ABCB4. In case of ABCG5/G8, it was suggested that bile acids promote an active conformation of ABCG5/G8 by acting as a chemical chaperone (48). In contrast to ABCB4, purified ABCG5/G8 did not show any ATPase activity in the presence of the natural substrate cholesterol. Only after adding bile acids, ATP hydrolysis was detected. The authors therefore concluded that bile acids act as a chemical chaperon. However, bile acids modulated the activity of both transporters at different concentrations. ABCB4 was stimulated in the μM range, while ABCG5/G8 becomes active at concentrations higher than 1 mM suggesting that micelles are required for the stimulation of ABCG5/G8. Therefore, a different mode of action of bile acids on both ABC transporters should be considered.

### Modulation of ATPase activity of ABCB4 in the presence of DOPC

It is now commonly accepted that ABCB4 only flops lipids of the PC family from the inner to the outer leaflet of the canalicular membrane of hepatocytes (2, 40). This study demonstrated that bile acids modulated the ATPase activity of ABCB4, which does not necessarily suggest that bile acids are a new family of substrates. To address this question, we analyzed the modulation of ATPase activity of ABCB4 in the presence of DOPC, cholesterol, and bile acids. In a previous study, it was shown that DOPC stimulates ATPase activity of human ABCB4 similar to a liver PC–lipid mixture (54). The effect of cholesterol and bile acid should be measured at maximal stimulation by DOPC, which corresponds to 50–60 μM. Thus, a concentration five to six times higher (300 μM) was chosen in our setup. The ATPase activity for maximal DOPC stimulation was set to 100% and modulation of cholesterol (Fig. 7) or bile acids (Fig. 8) as well as a combination of both (Fig. 9) was analyzed. A doubling of the ATPase activity was observed for cholesterol in a range of 300–600 μM (cholesterol to DOPC molar ratio ranging from 1:1 to 2:1, Fig. 7). Higher ratios resulted in a reduction to the initial value. Below a ratio of 1:2, no modulation was observed within experimental error. Thus, cholesterol is capable to enhance the stimulation beyond its maximal value induced by DOPC. Increasing amounts of cholesterol in purified membrane vesicles containing human ABCB11 (BSEP) resulted in a duplication of the ATP-dependent transport of different bile acids (47).

The modulation of ATPase activity of ABCB4 was also analyzed in the presence of DOPC and bile acids (Fig. 8). Glycine-conjugated versions of CA, CDCA, and DCA enhanced stimulation of the ATPase activity of ABCB4 in the presence of DOPC. In the case of taurine-conjugated bile acids, only TCA displayed a clear additional stimulation, while all others did not show an enhanced stimulation within experimental error. Interestingly changing the conjugation to a nonnatural conjugation as the one in CHAPS and CHAPSO resulted in a decrease of DOPC-stimulated activity by approximately 50%. Most importantly, Nor-UDCA revealed a higher increase of the DOPC-stimulated ATPase activity of ABCB4 than all other bile acids. ATPase activity was nearly four times higher if both were present Nor-UDCA and DOPC compared with only DOPC. Additionally, this stimulation remained the same if cholesterol was included in the analysis (Fig. 9B, brown bar) suggesting a new mode of action of Nor-UDCA. So far, the positive effects of Nor-UDCA were assigned to shortening of the biliary hepatic circulation, promoting a bicarbonate-rich choleresis, direct anti-inflammatory, antiproliferative, or antifibrotic effects (30). Here we demonstrate that unconjugated Nor-UDCA is capable of increasing the ATPase activity in the presence of the natural substrate DOPC. Although Nor-UDCA cannot be conjugated in the hepatocytes, glycine-conjugated Nor-UDCA may be considered in further studies, since we observed that glycine conjugation exceeded all other bile acids suggesting a new mode of interaction of ABCB4 and Nor-UDCA.

In a last approach, a BAM as close as possible to the in vivo situation was tested. Therefore, a BAM of GCDCA, TCDA, GCA, TCA, GDCA, and TDCA with a molar ratio of 21:19:24:16:13:7 as described (48, 77, 78) was investigated on ABCB4 (Fig. 9A) and in the presence of DOPC as well as in the presence of DOPC and cholesterol (Fig. 9B). The BAM stimulates ATPase activity of ABCB4 by ∼566% ± 17.6%. This is in the between the v_max_ values for individual G/TCDCA and G/TCA bile acids (Table 1), which make up the majority of this mixture. Therefore, the mixture stimulates ATP hydrolysis of ABCB4 similar to the individual bile acids. In contrast the EC_50_ value of the BAM is significant higher than for individual G/TCDCA and G/TCA kinetics. This may be to the fact that the specific concentration of individual bile acids in the mixture is 4–5 times lower. This also explains why v_max_ is reached at higher concentration (500–600 μM) of BAM compared with individual conjugated bile acids (Fig. 3). In the case of the BAM/DOPC sample (Fig. 9B, dark blue bar), an additional increase of 56% ± 26 compared with only DOPC (cyan bar) was detected, which is in the range of the individual bile acids (Fig. 8). Adding the BAM to the cholesterol:DOPC mixture (Fig. 9B, dark violet bar) revealed again an additional stimulation compared with only the cholesterol:DOPC mixture (Fig. 9B, dark gray bar). But the last approach including Nor-UDCA instead of BAM (Fig. 9B, brown bar) displayed the highest stimulation similar to Fig. 8. Here once more we observed that Nor-UDCA has the highest impact on ABCB4 ATPase activity and should be considered in future ABCB4 research.

In summary, we have demonstrated that ABCB4 is a PC–lipid translocase, whose ATPase activity is enhanced in the presence of bile acids and cholesterol. Obviously, this enhanced activity might result in an increased rate of lipid flop from the inner to the outer leaflet of the canalicular membrane. This clearly indicates an intricate cross talk of the substrates of the transporters of the bile triumvirate and their regulation to fulfill their physiological function.

## Data availability

All data are contained within the article.

## Conflict of interest

The authors declare that they have no conflicts of interest with the contents of this article.
